# From kill the winner to eliminate the winner in open phage-bacteria systems

**DOI:** 10.1371/journal.pcbi.1010400

**Published:** 2022-08-08

**Authors:** Anastasios Marantos, Namiko Mitarai, Kim Sneppen

**Affiliations:** Center for Models of Life, Niels Bohr Institute, University of Copenhagen, Copenhagen, Denmark; University of Chicago, UNITED STATES

## Abstract

Phages and bacteria manage to coexist and sustain ecosystems with a high diversity of strains, despite limited resources and heavy predation. This diversity can be explained by the “kill the winner” model where virulent phages predominantly prey on fast-growing bacteria and thereby suppress the competitive exclusion of slower-growing bacteria. Here we computationally investigate the robustness of these systems against invasions, where new phages or bacteria may interact with more than one of the resident strains. The resulting interaction networks were found to self-organize into a network with strongly interacting specialized predator-prey pairs, resembling that of the “kill the winner” model. Furthermore, the “kill the winner” dynamics is enforced with the occasional elimination of even the fastest-growing bacteria strains due to a phage infecting the fast and slow growers. The frequency of slower-growing strains was increased with the introduction of even a few non-diagonal interactions. Hence, phages capable of infecting multiple hosts play significant roles both in the evolution of the ecosystem by eliminating the winner and in supporting diversity by allowing slow growers to coexist with faster growers.

## Author summary

We demonstrate that in an open system of phages and bacteria with very limited resources, a bacterial strain that has a high growth rate can still be outcompeted by a slower-growing strain if they have a common phage. The impact of this on ecosystem structure is significant as soon as there is a small probability to have a common phage among bacterial strains. Furthermore, by analysing the structure of the interaction network we show that it self-organizes into a network with strongly interacting specialized predator-prey pairs, in order to reduce phages competition. Nevertheless, the presence of the remaining links is very important for the network dynamics since even a few of them significantly enhance the frequency of slower-growing strains.

## Introduction

The epipelagic oceanic zone (0–200 meters depth) is an open environment with sub-parts that have some influx of new phage and bacterial strains. Such an environment makes it very hard for bacteria to hide from phages, exposing them to direct predation. In fact, it has been estimated that phages kill 15–40 % of the ocean’s bacteria every day [1]. Despite that, phages and bacteria manage to coexist. This was quantified e.g. by the colossal work of Moebus and Nattkemper [2], where they collected water samples from many different locations in the Atlantic ocean and reconstructed a large microbial network of interacting phages and bacteria. An updated version of the study has been recently conducted by [3], confirming the patterns found in Moebus and Nattkermper data and further revealing additional information on the ecosystem from genome sequencing. Yet, how the phage-bacteria system can maintain diversity in such an open environment is still a standing question in ecology [4–9].

Two strains of bacteria (or phages) that fully share their resources (hosts) cannot coexist. This statement is known as the competitive exclusion principle [10–15], and more generally, the number of consumers that coexist cannot exceed the number of resources [16]. This principle reflects a detrimental competition between the fastest-growing strain and any slower-growing ones. Survival of the fastest grower and its tempting connection to the survival of the fittest [10] lead Fisher and his followers to use net growth rate as a measure of fitness [17]. This view was challenged by the “kill the winner” scenario of Thingstad [8] where steady-state populations are maintained by predators independent of their Malthusian growth rates. In steady state the net growth rate of all the species is zero, suggesting a neutral selection without fitness bias for further evolution. In this work we will augment this perspective, and show that basal Malthusian growth rate still plays a role for the long term survival.

The “kill the winner” scenario considers a system of multiple specialist phage-bacteria relations where all bacteria are limited by the same resource. In this scenario, the fastest-growing bacteria will be preferentially targeted by phage predation due to their large biomass and by suppressing their biomass, they leave space for slower-growing bacteria to enter the system and coexist.

This concept was extended to the sufficient condition for the coexistence of diverse bacteria strains and virulent phage strains by Haerter *et al*. [18]. They showed that irrespective of the phage-bacteria interaction network structure, the number of bacteria strains can exceed the number of phage strains at most by the number of independent resources for bacteria. As diversity increases the remaining resources diminish due to consumption by an increasing number of marginally abundant susceptible bacteria, resulting in “a narrowing staircase” of coexistence [18, 19]. To increase the diversity with a limited number of resources, the system needs to evolve with keeping the balanced diversity of phages and bacteria, which was in agreement with the experimental data [2, 20].

The above works revealed the conditions for the phage-bacteria interaction networks that allow coexistence. As example systems that satisfy the conditions, analyses in [18, 19] focused on the diagonal and nested organization of the phage-bacterial network. However, there are different networks that satisfy these conditions. It may be diagonal as in the “kill the winner model” [8], it may be nested [9, 20–22], or it may be more randomly organized. The actual ecosystem realized in nature should depend on the history of species assembly [21–24]. Having the phage-bacteria ecosystem in an open oceanic condition in mind, we here explore how the ecosystem assembles with a stochastic invasion of phages and bacteria by using dynamic models of a well-mixed microbial ecosystem. In particular, we quantify the importance of the bacterial growth rate as their fitness and how the phages can mediate the successful invasion of slow growers. We find that although the classical “kill the winner” suppression of the fastest grower is the norm, the fastest-growing bacteria typically exist longer than the slower grower. Despite that, occasionally a slow-growing bacteria completely eliminates a faster grower, reflecting true “kill the winner” events. These types of events also limit the lifetime of the fastest-growing bacteria without the need of assuming extinction by fluctuations in small population numbers [25].

## Methods

### Model

In our model, we analyze how the ecosystem naturally assembles in an open system where new bacteria and phage strains are introduced occasionally. For simplicity, we consider a well-mixed ecosystem of bacteria and virulent phages [8, 18, 20]. We assume that the resources are fully shared between all bacterial strains, i.e. if there are no phages, there will be only one bacterial strain that can stably exist in the environment; the fastest growing one. We consider macroscopic populations of strains and ignore latency as we focus on systems that reach steady-state before each new addition of a phage or bacterial strain. More specifically, for a given set of strains, the dynamics of bacterial (*B*_*i*_) and phage (*P*_*k*_) population densities are assumed to be governed by a set of generalized Lotka-Volterra equations [12, 18, 21, 26, 27]:

$$\begin{array}{c} \frac{dB_{i}}{dt}=k_{i}B_{i}(1-\sum_{j=1}^{N}p_{ji}B_{j})-\alpha B_{i}-B_{i}\sum_{k=1}^{M}\eta_{ki}P_{k}\\ \frac{dP_{k}}{dt}=P_{k}\sum_{m=1}^{N}\beta_{k}\eta_{km}B_{m}-\delta P_{k} \end{array}$$

The rate parameters are measured in units of the maximal possible bacterial growth rate and all the density parameters in units of the carrying capacity of the environment. *k*_*i*_ is the bacterial growth rate of strain *i* reported to be of order 2/day [28]. *α* = 0.1 is the universal death rate of bacteria (e.g. due to protist predation that is expected to amount to half bacterial death in the oceans [28]) and *δ* = 1 is the decay rate of phages (phage decay rates in the ocean is about 2/day [29]). *p*_*ji*_ represents interactions between bacterial strain *j* and bacterial strain *i*, and is here assumed to be purely competitive (*p*_*ji*_ > 0). *η*_*ki*_ is the infection rate of bacterial strain *i* by phage strain *k* whereas *β*_*k*_ is the burst size of the phage strain *k*.

We start our simulation with a system of only one bacteria strain with a growth rate selected uniformly ∈ [0, 1] in the steady-state of non-zero population size (this requires the growth rate bigger than *α*). Then, at each update step, we allow either a phage strain or a bacterial strain to invade the system. These additions are arbitrarily set to be equiprobable, but the qualitative messages of our work do not change substantially if invasion rates are asymmetric. A new strain is only allowed to invade when the population sizes are stabilized, which represents our next updating step. Thus our timescale corresponds to a series of events that lead to coexisting steady states of phage and bacteria strains.

We assign each bacterial strain a growth rate *k*_*i*_ selected uniformly ∈ [0, 1]. Moreover, *p*_*ji*_ = *p*_*ij*_ = 1 which reflects bacteria that interact directly by competing for a shared resource. *η*_*ki*_ ∈ (0, 1) is sampled randomly and is considered to be a characteristic of every phage-bacterial strain interaction, while *β*_*k*_ ∈ [1, 50] and is uniformly distributed and is a characteristic of each phage strain. Every new strain that is introduced to the system starts with a population density of 10^−6^.

In the actual computation of the steady-state of a given strain combination, we follow Haerter et al. [18] and consider linear steady-state equations ($\frac{1}{B_{i}}\frac{dB_{i}}{dt}=0$ and $\frac{1}{P_{k}}\frac{dP_{k}}{dt}=0$) to evaluate whether these linear equations provide non-degenerate and feasible (positive) solutions. If the system with a newly added strain yields a feasible steady-state solution, the strain is added and the system is updated to this new steady state. Each time an added strain implies that solutions do not fulfil these criteria, at least one strain is bound to go extinct. When this happens in our computational models, we need to identify which strains should be dropped out of the system. To do this we start from the steady-state populations of the previous time step and integrate the equations with the Runge–Kutta fourth-order method until one strain population falls below a very small threshold (10^−20^).

**Fig 1:**
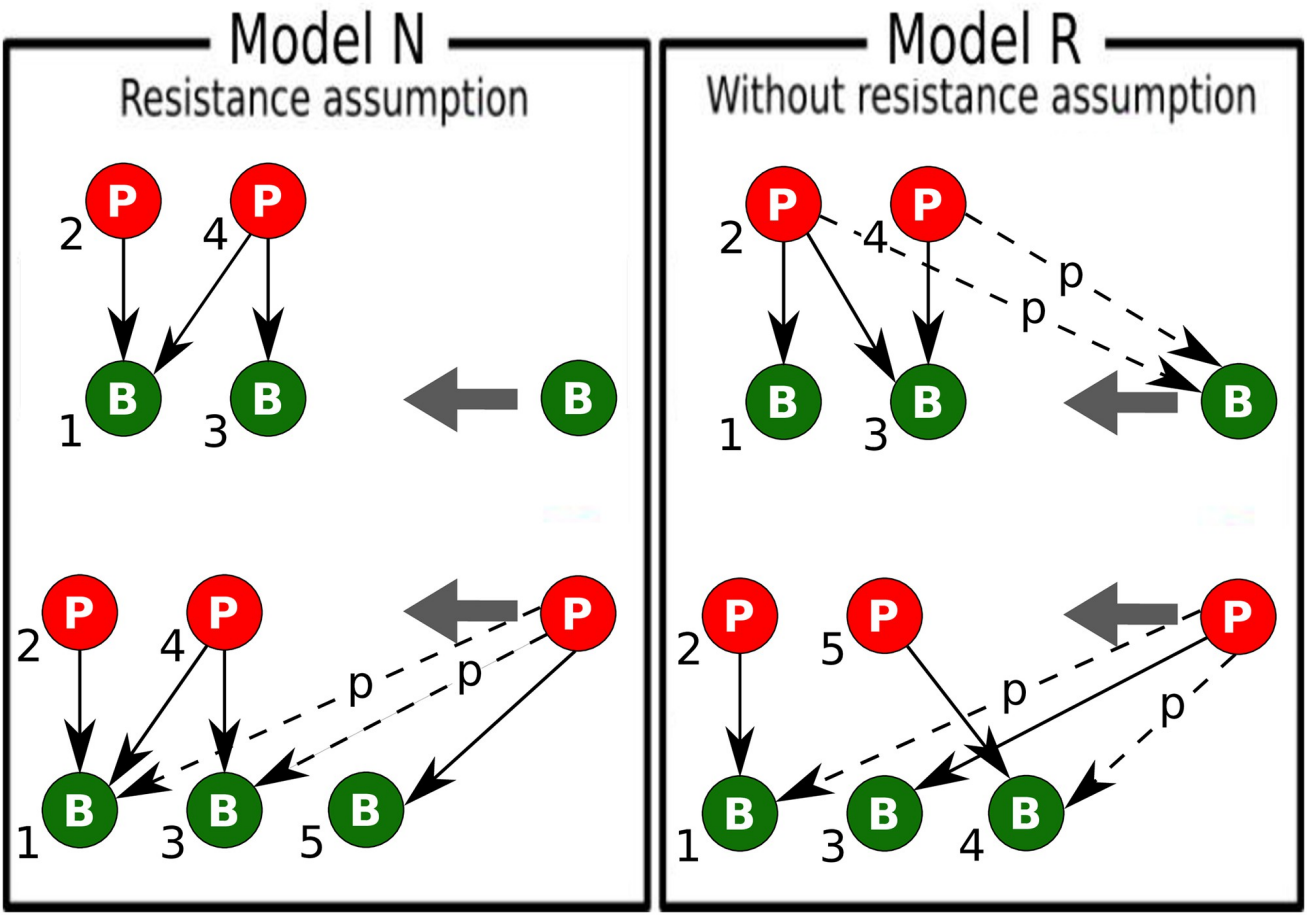
Schematic representation of Models N and R. The green (red) circles correspond to bacteria (phage) strains, hierarchically ordered from oldest to newest in the system (numbers). Notice that in model R, the new phage may attack a bacteria that is not the latest added. It just attacks whoever of the bacteria are not attacked by other phages (there can only be one of these). In model N the last added bacteria will automatically be the phage free one.

We consider two models for the possible phage-bacteria interactions with a newly added strain (Fig 1). Model N (nested by construction) is based on the assumption that only bacterial strains resistant to all existing phages can enter the system. The phages that try to invade the system attack the bacterial strain that does not have a phage predator and in addition every other bacteria with probability *p* ∈ [0, 1]. This choice reflects the dominant population of the “phage-free” bacteria.

Model N will organize the bacteria historically, with the oldest being potentially infected by all phages, while the younger are only exposed to recently introduced phages. The model N will produce a diagonal network when *p* = 0 and a fully nested network when *p* = 1, the two extremes of nested networks that were studied in [18]. Here we generalize by further allowing for any value of *p* which will again yield sustainable interactions for balanced diversity. One of the main reasons for the study of model N is that there is a general consensus in the community that marine phage-bacteria networks exhibit significant nestedness [2, 3, 8, 9, 18, 20–22].

In Model R (random assignment of attack) we relax the rule about nested order of attack. Thus here we allow any of the already existing phages to attack the newly introduced bacteria with some probability *p* ∈ [0, 1]. Further, as in model N, a new phage is set to attack a bacterial strain that doesn’t have predators with probability 1 and every other bacteria with probability *p* ∈ [0, 1]. The model R will produce a network with all possible bacteria-phage pairs connected when *p* = 1.

## Results

**Fig 2:**
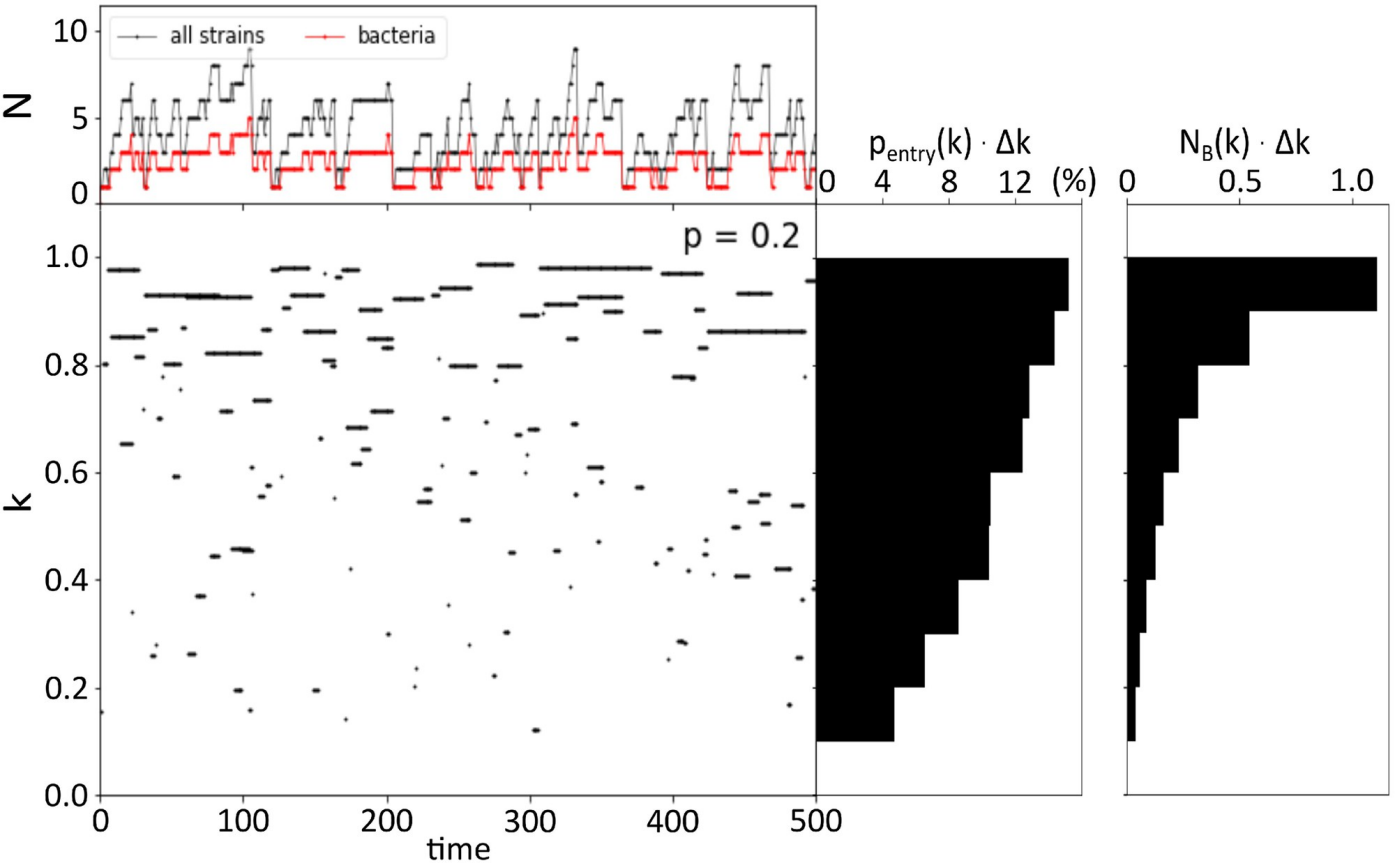
Evolutionary dynamics in Model R. The central plot shows the dynamic replacements of bacterial strains with time (cross-link probability *p* = 0.2). Each horizontal line corresponds to a bacterial strain, with the ordinate indicating its maximal growth rate. The top panel captures the number of bacterial strains (red line) and the total number of phages and bacteria (black line). The first right-hand panel displays *p*_*entry*_(*k*)Δ*k*, the distribution of the probability of a bacterial strain to enter the system as a function of its growth rate sampled over 10000 time-steps. The second right-hand panel shows *N*_*B*_(*k*)Δ*k*, the distribution of the average number of bacteria that exist at each time-step in the system as a function of their growth rate over 10000 time-steps. The bin size for sampling in *k*-space is taken to be Δ*k* = 0.1.

Fig 2 illustrates the dynamics of model R in time units of subsequent bacteria or phage invasion trials (see S1 Fig of the parallel plot for model N, which shows similar features). The central plot show existence of individual bacteria in terms of their growth rate.

The upper panel presents the vertical projection of the central plot in terms of the number of bacteria strains (red) and the total number of bacteria and phage strains (black). The fact that the total number of the strains is up to two times larger than the number of bacteria is in line with the balanced diversity manifested in the observational data analyzed by [18]. Also, the total number of strains is in agreement with the expectation from the narrowing staircase of the evolving “kill the winner” scenario of [18]. Furthermore, it is seen that even system’s size extinction events are possible, reflected in occasional collapses to only one strain.

The right-hand side panels constitute a horizontal projection of the central figure. The first one (starting from the left) shows the growth-rate dependent probability density *p*_*entry*_(*k*) of bacteria to enter the system, while the second plot shows the average number density *N*_*B*_(*k*) of bacteria that exist at every time-step as a function of their growth rate. It is immediately apparent that there is a systematic tendency to favour a high growth rate. The central plot corroborates that result, as one can see that bacteria with high growth rates tend to live longer. Thus, although “kill the winner” is at play on a population level, with populations of fast growers being suppressed by phages, there is some long term advantage to being a fast-growing strain. However, there are situations where the fastest growing strain can be driven to extinction by a slower-growing one.

**Fig 3:**
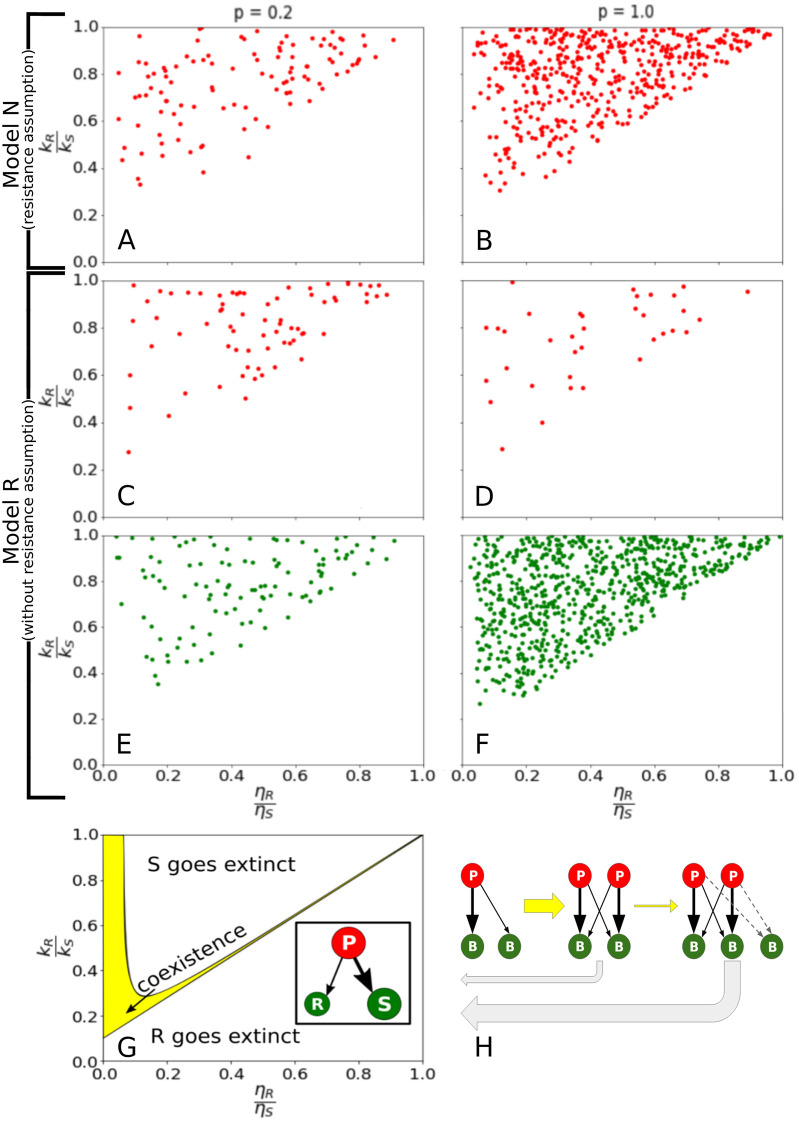
Extinction events. (A-F) Elimination of the fastest grower (S) by a slower-growing bacterial strain (R), using one common phage. The *x*-axis show ratio *η*_*R*_/*η*_*S*_ of infection rates to the bacterial strain, while *y*-axis marks the ratio *k*_*R*_/*k*_*S*_ of growth rates. Red dots mark events initiated by a phage while green dots mark events initiated by new a bacterial strain. (G) Analytically calculated regions of coexistence and extinctions for the above system (see S1 Appendix). The insert shows the motif with growth rates marked by circle size and susceptibility by the thickness of arrows. The yellow region marks the coexistence of all three strains. (H) Illustration of the evolution of the network as strains are added. Extinctions (grey arrows) are more likely as the system grows.

In order to understand the dynamics that govern those extinctions, we explore the network structure around strain extinction events. In Fig 3 we examine events where the fastest-growing bacteria (*S*) is replaced by a slower grower (*R*). We found that, when there is such an event, there is always a phage that infects both *S* and *R*. Therefore, the events are characterized by the ratio of the growth rate of the slow grower to the fast grower *k*_*R*_/*k*_*S*_ in the vertical axis and the ratio of the common phage’s infection rate to each strain *η*_*R*_/*η*_*S*_ in the horizontal axis. Each of these events is marked by a dot and was triggered by the invasion of a new strain in the system. The colour of the dots indicates the type of these invasive strains (red for phages and green for bacteria). In Model N (Fig 3A and 3B), it is always the introduction of a phage that initiates the extinction because a slow-growing bacteria cannot eliminate any faster-growing bacteria without help. In contrast, in model R, extinctions of fast growing bacteria can be initiated by both a phage or a bacteria that support an already present phage (Fig 3C–3F).

These patterns can be easily understood when we consider the simpler system of only two competing bacteria and see how the system’s diversity could possibly increase as a new strain is added to the system. In our single food source scenario, a faster and a slower-growing bacterium can only coexist with the help of a phage. Coexistence is then possible for a rather narrow set of parameters, provided that the slower grower is less exposed to the common phage than the faster grower (therefore represented as a bigger circle with S for faster-growing but more susceptible to phage attacks and a smaller circle with R for slower-growing but more resistant). This set of parameters is illustrated by the yellow area in Fig 3G [30, 31]. Remarkably, there is an even bigger range of parameters where the slower grower can out-compete the fastest grower and dominate the system. And of course, there is the biggest range of parameters where the fast-growing bacteria eliminates the slow grower (see S1 Appendix for the analytical calculation of these parameter regions). The striking similarity of the region where the faster growing but susceptible to the common phage goes extinct, between the Fig 3A–3F and 3G, constitutes a strong indication that the main mechanism through which a slower growing but resistant bacterial strain can out-compete a faster-growing yet susceptible to the common predator strain is through this triplet motif.

However, model N and R differ in the invasions that drive such extinctions. In Model N (Fig 3A and 3B), it is always the introduction of a phage that initiates the extinction, because of the assumption that the bacteria just entering the system are resistant to all existing phages. Therefore, the triplet with common phage can only be formed with the introduction of a phage strain that attacks both the faster-growing strain and the free, slow-growing bacteria that entered the system last before it.

On the other hand, extinctions in model R can be initiated by both a phage and a bacteria (Fig 3C–3F). When the probability *p* for connecting to existing strains is small, phages and bacteria invasions are equally likely to cause extinctions (see Fig 3C and 3E). However as *p* increases the extinction of the fastest grower is increasingly triggered by the invasion of a bacteria (Fig 3D and 3F). This reflects triplets formed by the bacteria that invade while having a link to an existing phage that is already preying on another bacteria. In a fully connected network as obtained for *p* = 1 in Model R, the probability for the formation of these triplet motifs is 1 already for total number of strains N = 3 and their number grows with the number of bacteria strains *N*_*bacteria*_ as $N_{bacteria}^{2}$ for *N* > 3. Therefore the closer our network is to the fully connected one, the higher the number of triplets that should be sustained (Fig 3H). As a result coexistence region becomes progressively narrower, setting practically an upper limit to the system’s size and an increase in the number of extinction events of the fastest growing bacterial strain.

**Fig 4:**
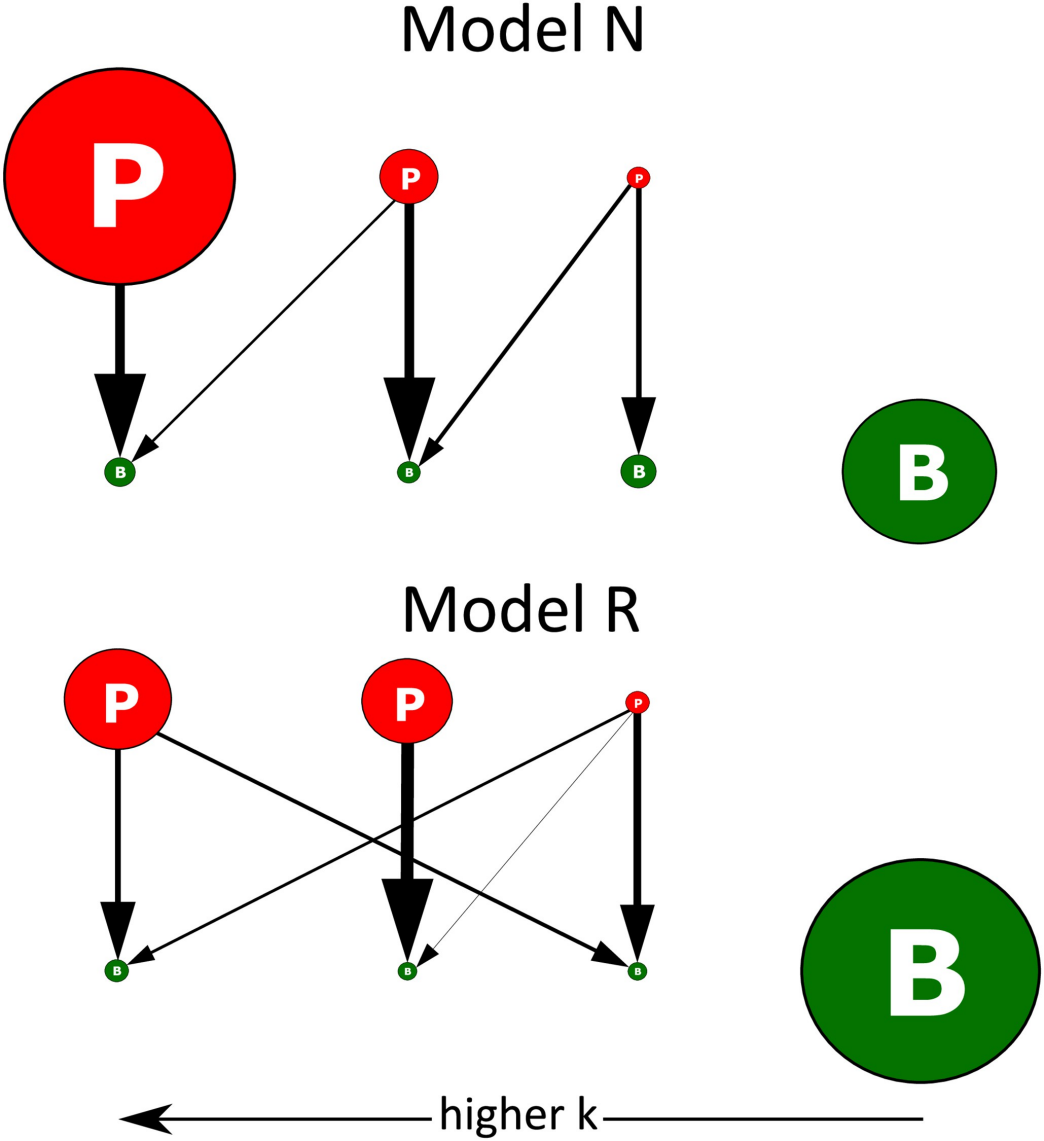
Example of interaction networks. The upper and lower panels show ecosystem examples for respectively the nested (N) and the random model (R). In model N the phage can only prey on bacteria that were present when the phage was introduced. This often concurs with the ordering after growth *k*, as older bacteria typically have larger *k*. In the model R an old phage can (with probability *p*) prey on a new bacteria which opens for the more random organization. Simulation is done with *p* = 0.5 and the sizes of the circles are proportional to the population. The width of the arrows is proportional to *βη* of the corresponding interaction. Notice the parallel strong links that give rise to phage-bacteria pairs regarding dominant interactions.

In addition to the replacement of the fastest grower, the detailed study of the network structure illuminates some interesting patterns in phage-bacteria ecology. In Fig 4, we show example networks from model N and model R at *p* = 0.5. Here, the link width is proportional to *βη*, which signifies the importance of the link. We see that both networks exhibit parallel strong links forming phage-bacteria pairs, even though a phage can infect multiple hosts and a host can be attacked by multiple phages. In other words, the predator-prey pairs are fairly specialized, reflecting a return to the “kill the winner” organization (where each strain had only one link).

**Fig 5:**
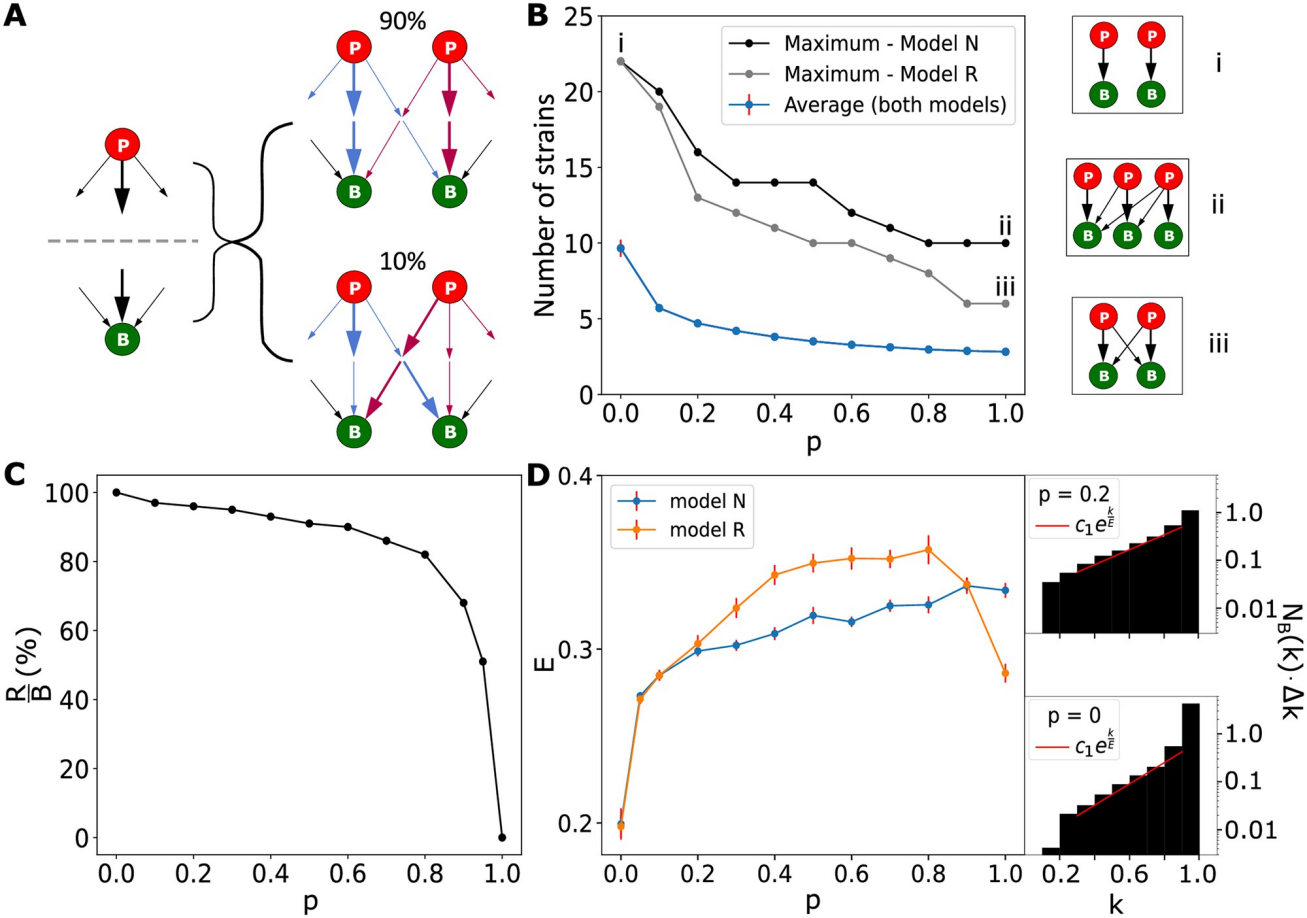
Analysis of evolved networks as a function of cross-link probability *p*. (A) Each phage (bacteria) has a number of links of different “strength” *βη*. In ∼90% of stable ecosystems, the strongest links (bold arrows) from phage and to bacteria coincide for all strains, forming pairs. Different arrow colors correspond to different phages. (B) Average and maximal diversity obtained for 10000 additions to the system. The sub-panels show characteristic networks at the corresponding points. (C) Analysing events in Model R where a new bacterial strain leads to increased diversity. The panel show the fraction of these events where the new bacteria are resistant to all preexisting phages. (D) The Elimination factor *E* of fast to slow growers as a function of *p*. The side panels show how the elimination factor is obtained from the scaling of steady-state diversity with the growth rate *k*. Both side panels correspond to model R.

We quantified how often this specialization is seen in our model (Fig 5A). We found that in about 90% of all stable ecosystems, all the strains form pairwise specialized predatory links that resemble the “kill the winner” structure. This means that stable ecosystems typically require phages that are disproportionately more adapted to infect the bacterial strain which is the most vulnerable to their attacks. This high prevalence of emergence of “kill the winner” systems was seen for both the N and the R model, decreasing from about 90% at *p* ∼ 0.1 to 80% at *p* ∼ 1. This close to the diagonal organization of predatory links in the bacteria-phage interaction matrix reflects a selection that minimizes similarity between phages and thereby reduces the competitive exclusion between the phages.

Fig 5B illustrates how the diversity decreases as the probability *p* of predatory links increases. In the *p* = 0 case where the interaction matrix is diagonal (See the subpanel Fig 5Bi), diversity is the highest because the role of the phages is limited to neutralizing bacterial competition. Hence, model N and model R are indistinguishable. As *p* increases the diversity decreases, reflecting a gradual change toward a system where all phages share the same resource (at *p* = 1) and competitive exclusion is only limited by different predation strengths of different phages (the *βη*’s). Furthermore, for larger *p* that maximal diversity in model N increasingly differs from that predicted by model R. This reflects the difference of the network structure at *p* = 1 for model N (subpanel Fig 5Bii) and model R (subpanel Fig 5Biii): more hierarchical predation increases phages’ differences, which in turn decrease competitive exclusion. This tendency can also be seen in Fig 4, where phages in model N have a clear hierarchy of population sizes, while in model R phages have similar population sizes to each other due to competition.

Competitive exclusion implies that a system with more phage strains than bacterial strains is unstable [18]. Therefore, a growing diversity needs an introduction of a new bacterial strain. Subsequently, such a new bacteria makes it relatively easy for a new phage strain to invade, provided that it is the main predator of that last bacterial strain. Fig 5C demonstrates that model R effectively behaves as model N, in the sense that successful invading bacteria that add to the diversity nearly always are resistant to all existing phages. Fig 5C shows the fraction R/B of such events where the invader is resistant to all phages. For small and moderate *p* values one sees that increase in diversity almost always relies on invaders having such universal resistance, i.e. *R*/*B* ∼ 1. First, for *p* exceeding 0.8, this fraction decreases. At *p* ∼ 1 any invading bacteria are set to be attacked by all phages in the system, and thus *R*/*B* is forced to be 0. This suggests that the “bacterial resistance as a prerequisite for entrance” assumption that was intrinsic to model N emerges as a consequence of also the more relaxed assumptions in model R. This also suggests that the results from the nested model simulations from Ref. [18] would be recapitulated by model R although with a smaller overall diversity.

Overall we have seen that the two models are indistinguishable for small *p*: They have the same upper diversity limits, and their diversity increases with the successful addition of a new bacterial strain, which subsequently allows for the addition of a new phage strain and the extinctions of their fastest-growing bacteria obey the same rule. Their difference only emerges for larger *p*, where invading bacteria to a larger extent drives diversity down, resulting in increased relative diversity for model R as *p* increases beyond 0.2.

The most interesting aspect of varying *p* is the fact that it modulates the classical “kill the winner” scenario associated with *p* = 0 to a situation where the slow-growing bacteria do even better when *p* is just slightly above 0. This is quantified in Fig 5D where we plot the elimination factor *E* of the fastest grower compared to the slower-growing bacterial strains. This is defined by the least square fit of

$$N_{B}\left(k\right)\propto \text{exp}(\frac{k}{E})$$

for *k* ∈ [0.3, 0.9], where *N*_*B*_(*k*) is the time-averaged number of bacteria as a function of the growth rate (see the right panels of Fig 5D for examples). A higher value of E represents relatively less diversity of faster bacteria compared to slower growers and correspondingly to a larger risk to be eliminated. Thus higher *E* represents an increased effect of eliminating the winner, while a low *E* ∼ 0.2 at *p* = 0 reflects the traditional “kill the winner” model. We see that already for *p* = 0.05, a sharp rise of *E* ∼ 0.28 is observed. This leads to a much more equal distribution of resources between bacteria with different sizes of growth speeds. This sharp mitigation of the advantage of the fastest growers with the slight probability for cross-link can also be seen in the entry to the system characterized by *p*_*entry*_(*k*) (cf. Fig 2), which is analyzed in the S2 Fig.

For *p* ≳ 0.8 Model R has markedly different behaviour from model N. This is seen in Fig 5C and 5D and S2 Fig. At such high *p* values the invading bacteria are attacked by most of the resident phages, and the few gains in diversity are often caused by invading bacteria that are susceptible to resident phages (Fig 5C). This makes differences between phages small and most parameters lead to eliminations by competitive exclusion. Furthermore, the phage load among bacteria becomes similar, and bacteria to a larger extent compete through their growth rate. As a consequence the maximal diversity decreases (Fig 5B) and the slope E drops (Fig 5D) for *p* above 0.8.

## Discussion

This work investigated open systems of phages of bacteria, mimicking a small patch of the ocean exposed to meandering phages and bacteria from outside the patch. Such sub-systems were sampled in the Atlantic ocean by [2, 32] and analyzed in [20] and [18]. Flores et al. [9, 20] highlighted the nested structure of the overall interaction network, while Haerter et al. [18] emphasized the balanced diversity of phages and bacteria at each local patch. By considering each patch as an open dynamical system we here studied the emerging structure and robustness of the local microbial networks.

Our model considers subsequent random invasions of individual phage strains or bacterial strains. We observe dynamics where any strain is exposed to extinctions; even the fastest-growing bacteria (Fig 2). Hence diversity of the developing one patch system occasionally collapses to one, thereby mimicking the wide range of diversities reported from a number of samples from the Atlantic ocean (see [2, 18, 33, 34]).

The elimination of even the fastest grower by a slower-growing strain separates our model from the standard “kill the winner” model. The growth speed of the fastest grower is not monotonic in time [18] or requires small populations eliminated by noise as suggested by [25]. Furthermore, the term “kill” by Thingstad [8] refers to repression in numbers that leads to coexistence, while our analysis demonstrates the pronounced effect of occasional elimination of the fastest grower. In other words, while the growth rate-based fitness measure in the Fisher sense [17] is dynamically driven to a maximum value, this optimal is punctuated when a common predator exposes the system to extreme consequences of apparent competition [5]. Fig 3 demonstrated the basic motif for this elimination of fast growers [5, 30, 31], highlighting the requirement of a weaker phage exposure (lower *η*) for becoming a winner in spite of being a slower grower.

The concept of using phages as a weapon has been investigated previously by [35], where an immune strain with a prophage was using phages from spontaneous induction events to invade a sensitive strain. Thereby a temperate phage was able to lead to a population redistribution that only could be maintained if lysogens of the two strains had the same growth rate. In fact, even if the sensitive strain was always lysed by infections one would typically end in a “kill the winner” type coexistence between a slower-growing lysogen and the faster growing sensitive strain.

Our evolving systems with virulent phages exhibit lower diversity when the apparent competition is increased (larger *p* in Fig 5B). This is what naively should be expected because higher *p* tends to diminish differences between predators, thereby increasing competitive exclusion. Remarkably, an increase in the frequency *p* of phage-bacteria interactions still leads to systems where each phage has a dominant host, and the host in turn is mainly predated by this same phage. In effect, the observed systems self-organise to resemble the diagonal “kill the winner” network supplemented with weaker cross-links (Fig 5A and 5C). This specialization emerges in order to minimize the competition between the phages and avoid competitive exclusion. This is different from the reasoning in classic trade-off theory, where specialization in interaction arises because it is costly for bacteria to sustain resistance to past phages and for phages to attack rare hosts [36, 37].

Perhaps the most remarkable aspect of cross-links is that they greatly favour the weak growers. Allowing phages to have more hosts, the pressure on the fast grower enhances and leads to a substantially larger diversity of the slow growers (Fig 5D and S2 Fig). The robustness of this effect is seen by the change in relative abundance when changing the model from the “kill the winner” scenario [8] with an implicit *p* = 0 to an “Eliminate the winner” model with a remarkably small but finite *p* ∼ 0.05.

From a wider perspective, this paper investigated how the structure of microbial networks depends on the rules at which they are assembled [21–24]. We demonstrated that the dominant interaction structures in the obtained networks were robust to the assumption of whether additions were specialized, nested or random. We further found that the total diversity decreased with the likelihood that newly added species interact directly with resident species. Importantly, the most general model (R) obtained nested microbial networks as an indirect effect of competitive exclusion against invading bacteria. Nestedness and specialization may thus emerge in an open system, without the need for an evolution constrained by mutating genomes in a more closed system. That such evolutionary dynamics lead to similar network structures was demonstrated by [38].

Overall, predators are regulators of diversity already in the original “kill the winner” model [8], and mediators of competition already in the original “apparent competition” model [5]. But here they also put limits on any absolute measure of fitness in terms of reproductive rate. They are the mechanism for killing the winner at a new scale, where the winner is really eliminated and a new race for a new fastest grower can be restarted. Therefore evolution/replacement dynamics proceed in waves [39], often restarting when the fastest grower is replaced.

## Supporting information

S1 Appendix Analytical calculations of Fig 3G. (PDF)

S1 Fig Evolutionary dynamics in Model N. The central plot shows the dynamic replacements of bacterial strains with time (cross link probability *p* = 0.2). Each horizontal line corresponds to a bacterial strain, with the ordinate indicating its maximal growth rate. The top panel captures the number of bacterial strains (red line) and the total number of phages and bacteria, (black line). The first right hand panel displays the distribution of the probability of a bacterial strain to enter the system as a function of its growth rate sampled over 10000 time-steps. The second right hand panel shows the distribution of the average number of bacteria that exist at each time-step at the system as a function of their growth rate over 10000 time-steps.
(TIF)

S2 FigEntry advantage related to growth rate.The central plot shows the relative entry advantage *G*_*A*_ of the fastest grower compared to the slower grower. The right subpanels display the definition of *G*_*A*_ as $G_{A}=\frac{slope}{P_{entry}\left(k=0.3\right)\Delta k}$ from the fitting of the distribution of the probability that a bacterial strain of growth rate k invades the system successfully. Both side panels correspond to model R but they would be practically the same for model N as one can see from the central panel.(TIF)
