# Supplementary material for: From kill the winner to eliminate the winner in open phage-bacteria systems
Source: PLoS Comput Biol. 2022 Aug 8;18(8):e1010400. doi: 10.1371/journal.pcbi.1010400 (PMC9387927; doi:10.1371/journal.pcbi.1010400)
Supplement: S1 Appendix — (PDF) [file pcbi.1010400.s001.pdf]

From kill the winner to eliminate the winner in open phage-bacteria systems

Anastasios Marantos[1], Namiko Mitarai[1], Kim Sneppen[1*]

1 Center for Models of Life, Niels Bohr Institute, University of Copenhagen, Copenhagen, Denmark

* ksneppen@gmail.com

Supplementary

S1 Appendix: Analytical calculations of Fig 3G

Our basic 3 node motif for elimination of faster growing bacteria can be expressed as:

$$\begin{aligned}\frac{dB_S}{dt} &= k_S B_S(1 - B_S - B_R) - \alpha B_S - \eta_S P B_S \\ \frac{dB_R}{dt} &= k_R B_R(1 - B_S - B_R) - \alpha B_R - \eta_R P B_R \\ \frac{dP}{dt} &= \beta(\eta_S B_S + \eta_R B_R)P - \delta P\end{aligned}$$

where β is burst size of phage, that for simplicity are assumed the same for each infection. Assuming that both bacterial strains co-exist ($B_R, B_S > 0$) one obtain for the steady state:

$$\begin{aligned}1 - B_S - B_R &= (\alpha + \eta_S P)/k_S \\ 1 - B_S - B_R &= (\alpha + \eta_R P)/k_R\end{aligned}$$

Thus coexistence require:

$$\begin{aligned}(\alpha + \eta_S P)k_R &= (\alpha + \eta_R P)k_S \Rightarrow \\ P &= \frac{\alpha(1 - \frac{k_R}{k_S})}{\eta_S(\frac{k_R}{k_S} - \frac{\eta_R}{\eta_S})}\end{aligned}$$

For $P > 0$ and $\frac{dP}{dt} = 0$ we get:

$$\begin{aligned}\beta(\eta_S B_S + \eta_R B_R) - \delta &= 0 \Rightarrow \\ B_S = \frac{\delta}{\beta\eta_S} - \frac{\eta_R}{\eta_S} B_R\end{aligned}$$

and as a result $B_R = 1 - B_S - \eta_S P - \alpha$ becomes:

$$B_R = 1 - (\frac{\delta}{\beta\eta_S} - \frac{\eta_R}{\eta_S} B_R) - \eta_S(\frac{\alpha(1 - \frac{k_R}{k_S})}{\eta_S(\frac{k_R}{k_S} - \frac{\eta_R}{\eta_S})}) - \alpha$$

Therefore, at the steady state we have:

$$B_S = \frac{\delta}{\beta\eta_S} - \frac{\frac{\eta_R}{\eta_S}}{1-\frac{\eta_R}{\eta_S}}\left(1 - \frac{\delta}{\beta\eta_S} - \frac{\alpha(1-\frac{\eta_R}{\eta_S})}{(\frac{k_R}{k_S}-\frac{\eta_R}{\eta_S})}\right) \quad (1)$$

$$B_R = \frac{1}{1-\frac{\eta_R}{\eta_S}}\left(1 - \frac{\delta}{\beta\eta_S} - \frac{\alpha(1-\frac{\eta_R}{\eta_S})}{(\frac{k_R}{k_S}-\frac{\eta_R}{\eta_S})}\right) \quad (2)$$

$$P = \frac{\alpha(1-\frac{k_R}{k_S})}{\eta_S(\frac{k_R}{k_S}-\frac{\eta_R}{\eta_S})} \quad (3)$$

(3) implies that ($P > 0$) is only possible when $k_S > k_R$ and $k_R/k_S > \eta_R/\eta_S$). I.e. that the ratio of the ratio of growth rates exceeds the ratio of susceptibilities. But in order to have at the same time $B_S > 0$ and $B_R > 0$ we need:

$$(2) \Rightarrow \frac{\delta}{\beta\eta_S} + \frac{\alpha(1-\frac{\eta_R}{\eta_S})}{(\frac{k_R}{k_S}-\frac{\eta_R}{\eta_S})} < 1$$

$$(1) \Rightarrow \frac{\delta}{\beta\eta_S} > \frac{\frac{\eta_R}{\eta_S}}{1-\frac{\eta_R}{\eta_S}}\left(1 - \frac{\delta}{\beta\eta_S} - \frac{\alpha(1-\frac{\eta_R}{\eta_S})}{(\frac{k_R}{k_S}-\frac{\eta_R}{\eta_S})}\right)$$

$$\textit{that combined give}: \Rightarrow \frac{k_R}{k_S} > \alpha + (1-\alpha)(\frac{\eta_R}{\eta_S}) \quad (4)$$

$$\textit{and}: \Rightarrow \frac{k_R}{k_S} < \frac{\eta_R}{\eta_S} + \frac{\alpha\frac{\eta_R}{\eta_S}}{\frac{1-\frac{\delta}{\beta\eta_S}}{1-\frac{\eta_R}{\eta_S}} - 1} \quad (5)$$

and of course, by construction:

$$\frac{k_R}{k_S} < 1 \quad (6)$$

$$\frac{\eta_R}{\eta_S} < 1 \quad (7)$$

The inequalities (4),(5),(6),(7) define the region of coexistence in Figure 3G.

For $1 > \frac{k_R}{k_S} > \frac{\eta_R}{\eta_S} + \frac{\alpha\frac{\eta_R}{\eta_S}}{\frac{1-\frac{\delta}{\beta\eta_S}}{1-\frac{\eta_R}{\eta_S}} - 1} \Rightarrow P > 0, B_S < 0, B_R > 0$ implying that the slower growing but more resistant to phage attacks bacterial strain outcompetes the faster growing but susceptible in this parameter region with the help f the phages.

For $\alpha + (1-\alpha)(\frac{\eta_R}{\eta_S}) > \frac{k_R}{k_S} > \eta_R/\eta_S \Rightarrow P > 0, B_S > 0, B_R < 0$ meaning that despite the "help" from the phages, the resistance and growth of B_R is not high enough compared to B_S and it is driven to extinction.

For $\frac{k_R}{k_S} < \eta_R/\eta_S < 1 \Rightarrow P < 0, B_S > 0, B_R < 0$. The phages cannot survive. As a result B_R is also driven to extinction, as dictated by the competitive exclusion principle.
