# Supplementary figures and images for: From kill the winner to eliminate the winner in open phage-bacteria systems
Source: PLoS Comput Biol. 2022 Aug 8;18(8):e1010400. doi: 10.1371/journal.pcbi.1010400 (PMC9387927; doi:10.1371/journal.pcbi.1010400)

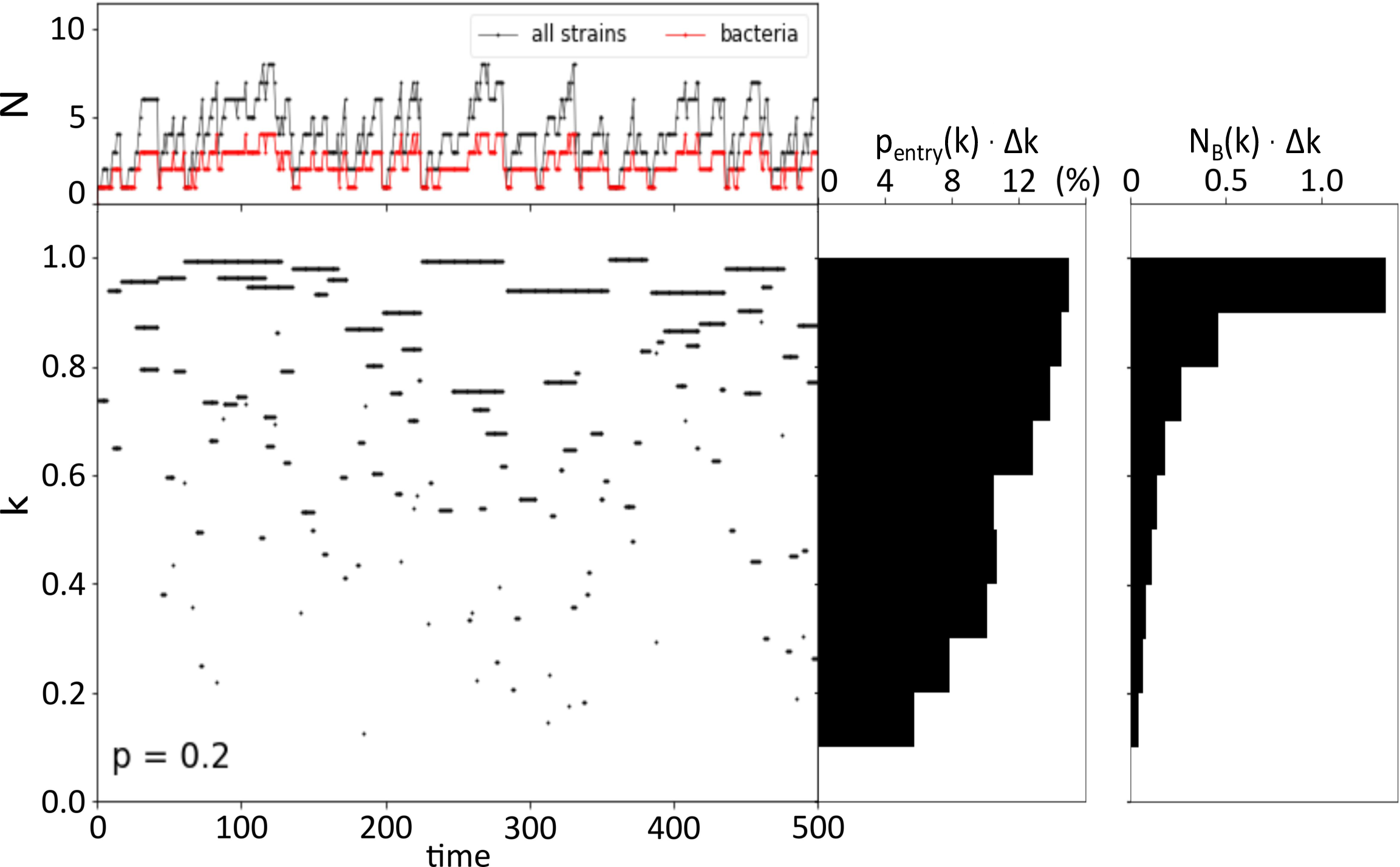

Supplement: S1 Fig — The central plot shows the dynamic replacements of bacterial strains with time (cross link probability p = 0.2). Each horizontal line corresponds to a bacterial strain, with the ordinate indicating its maximal growth rate. The top panel captures the number of bacterial strains (red line) and the total number of phages and bacteria, (black line). The first right hand panel displays the distribution of the probability of a bacterial strain to enter the system as a function of its growth rate sampled over 10000 time-steps. The second right hand panel shows the distribution of the average number of bacteria that exist at each time-step at the system as a function of their growth rate over 10000 time-steps. (TIF) [file pcbi.1010400.s002.tif]

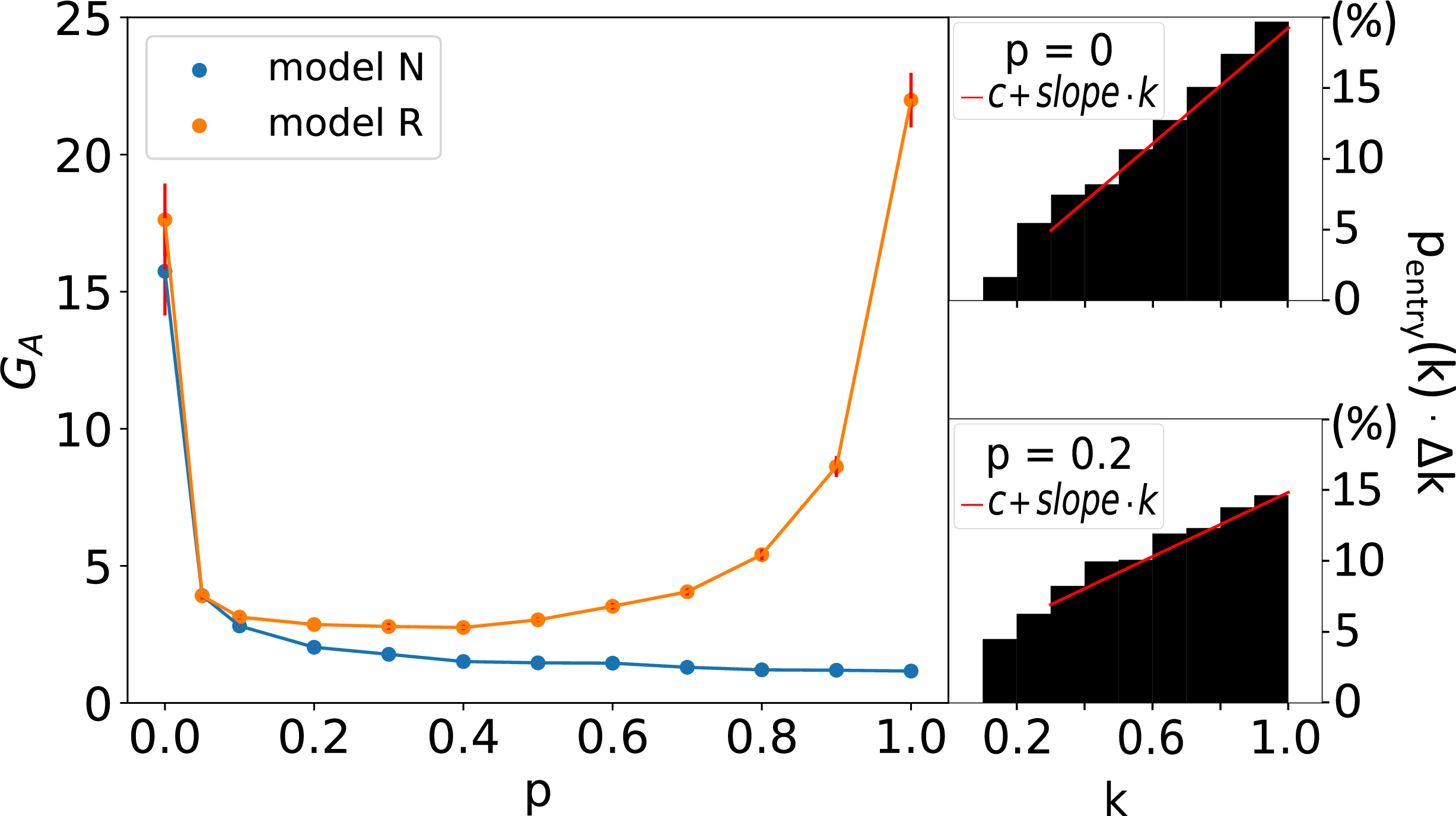

Supplement: S2 Fig — The central plot shows the relative entry advantage GA of the fastest grower compared to the slower grower. The right subpanels display the definition of GA as GA=slopePentry(k=0.3)Δk from the fitting of the distribution of the probability that a bacterial strain of growth rate k invades the system successfully. Both side panels correspond to model R but they would be practically the same for model N as one can see from the central panel. (TIF) [file pcbi.1010400.s003.tif]
